# Accuracy of Full-Arch Intraoral Scans Versus Conventional Impression: A Systematic Review with a Meta-Analysis and a Proposal to Standardise the Analysis of the Accuracy

**DOI:** 10.3390/jcm14010071

**Published:** 2024-12-27

**Authors:** Paolo Pesce, Paolo Nicolini, Vito Carlo Alberto Caponio, Piero Antonio Zecca, Luigi Canullo, Gaetano Isola, Domenico Baldi, Nicola De Angelis, Maria Menini

**Affiliations:** 1 Dental Unit, Department of Surgical Sciences (DISC), University of Genoa, 16132 Genova, Italy; paulnick99@outlook.it (P.N.); domenico.baldi@unige.it (D.B.); n.deangelis74@gmail.com (N.D.A.); maria.menini@unige.it (M.M.); 2 Department of Clinical and Experimental Medicine, University of Foggia, 71122 Foggia, Italy; vitocarlo.caponio@unifg.it; 3 Department of Medicine and Technical Innovation, Insubria University, 21100 Varese, Italy; pieroantonio.zecca@uninsubria.it; 4 Unit of Periodontology, Department of General Surgery and Surgical-Medical Specialties, School of Dentistry, University of Catania, 95124 Catania, Italy; gaetano.isola@unict.it

**Keywords:** digital impression, full-arch, dental implants, accuracy, dental impression technique, implant supported dental prosthesis, systematic review, meta-analysis

## Abstract

**Objectives:** The aim of this study was to systematically revise the state of art of the accuracy of digital and conventional impressions in clinical full-arch scenarios. **Methods:** Electronic and manual searches were conducted up to December 2024. Only trials comparing the accuracy of digital versus conventional impressions were selected by two independent reviewers. Accuracy was evaluated by analysing the fit of the prostheses obtained through conventional workflows and those obtained from digital workflows using intraoral scanners. Alternatively, accuracy was assessed by comparing the standard tessellation language data acquired from intraoral scanning with those obtained from scanning the physical model. The risk of bias was assessed using the Quality Assessment of Diagnostic Accuracy Studies tool. Meta-analysis was conducted to pool the mean differences from the included studies, with heterogeneity tested by Cochran’s Q test and quantified by the I^2^ index. **Results:** We included 9 relevant studies from a total of 2535 identified studies. The risk of bias was evaluated as low, and the main results of all the included articles reported similar accuracy between digital and conventional impressions. Random effects meta-analysis resulted in a pooled mean difference of 152.46 (95% C.I. = 76.46–228.46, *p*-value < 0.001, I^2^ = 93.48%). **Conclusions:** In conclusion, the results of the present systematic review reveal contradictory findings regarding the accuracy of digital impressions. However, most studies analysing the clinical performance of prostheses obtained through digital impressions suggest that their accuracy falls within clinically acceptable thresholds. Future research should report comparable outcomes and focus attention on linear deviations, comparing differences between conventional and digital impressions not in absolute terms, but relative to the distance measured.

## 1. Introduction

An accurate oral impression is decisive to fabricate a dental prosthesis with an ideal fit. Misfitting prostheses could lead to technical and biological complications and may hinder the long-term success of dental implants [1]. Among the technical complications there may be fractures of various components in the implant system (screw loosening, screw fracture, implant fracture), while pain, soft tissue inflammation, marginal bone loss, occlusal inaccuracy and even loss of osseointegration are among the biological issues [2,3,4]. Taking impressions is a fundamental step in obtaining accurate models for fabricating prostheses. In this regard, precision is crucial to ensure the proper fit and configuration of the prosthesis.

Unlike prostheses supported by natural teeth, where a range of movement of 100 μm is allowed, implant movements are much smaller and limited to 10 μm, so a very accurate prosthesis is necessary [4,5].

Digital workflow, thanks to the recent developments in computer-aided design and computer-aided manufacturing (CAD-CAM) technology in implant dentistry, has allowed the overcoming of some limitations of conventional impression techniques, primarily related to patient comfort. In fact, the process of taking a traditional impression can be unpleasant for some patients, such as children or individuals with strong gag reflexes. Conventional impressions are made using materials such as alginate, silicone and polyether, which often face issues with dimensional stability. Moreover, procedural errors like bubble formation, impression stretching and contact between the impression tray and teeth during various stages can compromise the accuracy of the result [6].

The use of digital impressions (DIs) and intraoral scanners (IOSs) can overcome these problems and offer advantages such as increased time efficiency. Beyond reducing chairside time, intraoral scanning also saves time in subsequent phases by eliminating the need for storage and transportation to the dental laboratory and the pouring of models [7,8].

Accuracy, according to the International Organization for Standardization (ISO), is a combination of trueness and precision. Trueness is defined as the degree of agreement between the value measured and the real dimensions of the object, while precision is defined as the variation between different scans of the same object [3].

Higher trueness means the scan closely resembles the object being scanned, while higher precision indicates that repeated scans produce consistent results. Trueness refers to the closeness of measurements to the actual values, and precision refers to the consistency of multiple repeated measurements [9,10].

Several studies analysed the in vivo accuracy of full-arch impressions on non-edentulous patients [11] or the in vitro accuracy of IOS in full-arch implant impressions, which is one of the most challenging clinical situations due to the absence of anatomic landmarks compared to partial edentulism [1,8,12,13,14,15,16,17,18].

On the contrary, in vivo research on the topic is limited. Therefore, the purpose of the present study was to systematically revise the literature on the accuracy of digital versus conventional impressions in clinical full-arch scenarios.

## 2. Materials and Methods

The present review was created following the PRISMA guidelines [19,20] and the review protocol was registered on the International Prospective Register of Systematic Reviews—PROSPERO (submission No. 42023494850).

### 2.1. Eligibility Criteria

A focused question was created according to the PICO format: Which is the accuracy of full-arch intraoral scans compared to traditional impressions in implant-supported full-arch rehabilitated patients? The PICO format comprises the following elements:

Population (P): patients rehabilitated with full-arch implant-supported prostheses;

Intervention (I): intraoral scan of the dental arch;

Comparison (C): conventional impression of the dental arch;

Outcome (O): accuracy.

Since these are the two most common methods to measure accuracy, accuracy had to be evaluated by one of the two following described methods. The first method was indirect and involved comparing two prostheses: one obtained by digital workflow and the other by traditional workflow. Accuracy was assessed using the Sheffield test and radiographic analysis to detect any gaps. The second method involved analysing and comparing the STL data from both workflows, focusing on distance and angular deviations, as some researchers might have presented their results based on linear deviation or on 3D root mean square (RMS) deviation.

This meta-analysis solely included clinical studies that met the specified criteria for inclusion: (1) clinical trials and observational studies; (2) human studies; (3) studies comparing intraoral scanning and conventional impressions; and (4) the prosthetic rehabilitation of at least four implants.

In contrast, articles were excluded if they met any of the following criteria: (1) duplication of prior trial reports; (2) lack of full-text availability; (3) case reports; (4) animal studies; (5) in vitro studies; (6) systematic reviews; or (7) meta-analyses.

### 2.2. Eligibility Criteria and Search Strategy

An electronic research was performed on four databases: Medline (PubMed), Scopus, the Cochrane Central Register of Controlled Clinical Trials (CENTRAL) and Web of Science (WOS). The last search was conducted in December 2024. The following search strategy was used on PUBMED and adapted for each database: (“Dental implants” [MeSH]) AND ((intra-oral impression) OR (IOS) OR (digital impression) OR (intraoral scanner)). Additionally, a manual search was conducted of the reference lists of the included studies and systematic reviews on the topic, which were carefully examined for the presence of additional studies to include. No restrictions were applied regarding the date of publication, but only articles written in English were selected.

Two authors (PN and PP) screened the titles, abstracts and full texts and Cohen’s Kappa statistic was employed to evaluate the inter-examiner agreement. In instances of uncertainty, a third co-author (VCAC) was consulted. The full texts of all eligible articles were obtained, and any exclusions were accompanied by documented reasons for their omission.

The data extraction process was carried out by two authors (PN and PP) using a Microsoft Excel spreadsheet. The extracted information included the year and journal of publication, authors, title, study design, IOS employed, material for conventional impression, sample size, number of implants, prosthesis fit and values of accuracy.

### 2.3. Risk of Bias Assessment

Two authors (PN, PP) independently evaluated the studies for the risk of bias. The assessment of risk of bias was conducted using the Quality Assessment of Diagnostic Accuracy Studies (QUADAS-2) tool. This tool evaluates four domains: patient selection, index test, reference standard, and flow and timing. Each domain is assessed for the risk of bias, while the first three domains are also evaluated for concerns regarding applicability. Signalling questions are included to aid in judging the risk of bias.

### 2.4. Synthesis Methods

The meta-analysis was conducted using ProMeta3. To pool the mean differences from the included studies, heterogeneity was tested by Cochran’s Q test and quantified by the I^2^ index. A fixed or a random effects model was employed to summarise the mean differences, standard deviations and the sample size of the included studies, based on I^2^ values. For values below 50%, a fixed effects model was employed to plot studies in a forest plot; otherwise, a random effects model was chosen.

## 3. Results

### 3.1. Study Selection

Exploring online databases, such as Cochrane (*n* = 163), Medline (*n* = 588), SCOPUS (*n* = 1031) and WOS (*n* = 753), led to the discovery of 2535 pertinent articles.

**Figure 1:**
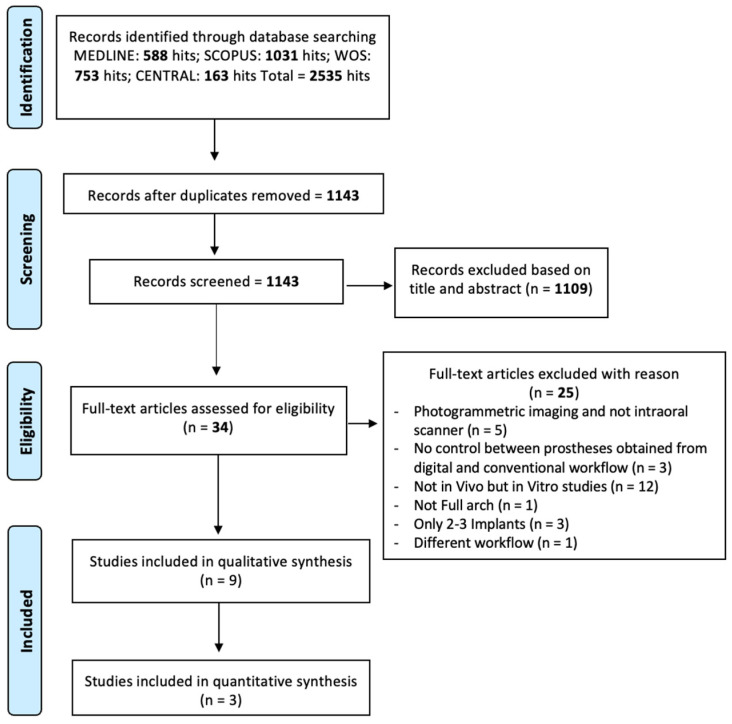
Flow-chart of the included studies.

After removing duplicates, 1143 articles underwent evaluation. Among these, 1109 were excluded during the title or abstract screening phase as they did not meet the inclusion criteria. The remaining 34 articles underwent full-text reading, resulting in the exclusion of 25 additional papers. The kappa value for inter-reviewer agreement was 0.975, indicating very good agreement. Nine studies were included in the systematic review and threest in the meta-analysis. The selection process is visually depicted in Figure 1.

### 3.2. Study Characteristics

Two main methods were used for accuracy assessment. Five studies investigated the accuracy of IOS, analysing the fit of the prosthesis obtained through conventional workflow and the fit of the prosthesis obtained from digital workflow [4,21,22,23,24]. The prosthetic fit was assessed with the Sheffield test and with radiographic examination. The main data are reported in Table 1.

Pera et al. [24], using the same cohort of nine patients and 11 dental arches, obtained both a traditional impression and a digital one. The traditional impression was made by an open tray technique with pick-up copings and plaster. The digital one was obtained using the Mach 2 IOS (Mach2 Intraoral Scanner Shining 3D, distributed by Euromax Monaco). The fit, precision and passivity of the substructures were clinically analysed through two criteria: the Sheffield test and radiographic examination. In the Sheffield test, the framework was deemed passive when intraorally tightening the screw on the distal abutment (using a dynamometric screwdriver with a tightening torque of 10 Ncm) did not result in a gap at the other framework–implant interfaces. Periapical radiographs, taken with the parallel technique after tightening all the prosthetic screws, were used to evaluate the marginal fit of the frameworks screwed onto the implants. The Sheffield test showed that both the digital and analogue methods produced frameworks with excellent passivity. In 81.81% of the cases (9 out of 11), the substructures had a perfect fit, while in 18.19% (2 out of 11), there was a slight discrepancy. Radiographic examination confirmed 100% accuracy, with no gaps between the frameworks and the implant heads or multiunit abutments.

Roig et al. [4] in a study similar to the previous one, analysed, in a cohort of 12 patients, the fit of a zirconia framework obtained with a conventional technique (impregum impression; 3M ESPE) or digital impression (TRIOS3; 3Shape). A prefabricated auxiliary device was used to adjust the IOS. The prosthesis fit was assessed according to five criteria: the perception of passivity during the insertion of the prosthodontic screws, tactile perception, radiographic examination findings, Sheffield test results and tightening torque. They concluded that prostheses produced using the completely digital workflow exhibited a better clinical fit compared to those obtained with the conventional workflow.

Gherlone et al. [22] and De Angelis et al. [21] analysed the results of digital and conventional impressions in two different cohorts of patients.

Gherlone et al. [22] analysed the fit of 30 frameworks, 15 realised with a conventional technique (Permadyne, 3M ESPE) and 15 realised with a digital impression (TRIOS; 3Shape). The fit was assessed clinically and radiographically. No difference was identified between the digital and the conventional group. Additionally, this study showed greater efficacy of the digital workflow than the conventional technique in terms of timing, patient satisfaction, a reduced likelihood of impression size variation and 3D previsualisation.

De Angelis et al. [21] compared the fit of 50 prostheses obtained through conventional impression (polyvinilsiloxane) with 50 prostheses obtained with digital impression (TRIOS; 3Shape). All the prostheses were considered clinically acceptable and immediately delivered without the need to take additional impressions. The fit was assessed radiographically and a statistically significant difference was identified among the conventional vs. digital groups, with better results in the latter one.

Additionally, four studies compared STL data acquired from IOS with those obtained from scanning the physical model [4,25,26,27], produced by conventional techniques (with polyether or PVS), using a laboratory scanner. The STL files of the two virtual models were superimposed by using a reverse engineering software program to measure the 3D coordinate system. The analyses included linear deviations, angular deviations and 3D RMS.

Moreover, Carneiro Pereira et al. [27] compared three impression techniques: digital scan bodies (group SC), digital scanning with a scanning device (group SD) and laboratory scanning of casts from splinted impression copings (control group CT). A linear displacement analysis was conducted, and the results showed that group SD performed similarly to the control group CT, whereas the SC group showed the highest values. The study concluded that the scanning device (group SD) provided improved accuracy for linear and angular displacements, as well as distances between implants in mandibular edentulous arches.

Also, Fu et al. [28] compared three techniques: IOS with prefabricated aids, conventional technique and photogrammetry. The distance and angle deviations between all pairs of abutment analogues and chairside time were measured. They concluded that the accuracy of photogrammetry and IOS with prefabricated scan aids were both clinically comparable. Additionally, the inter-abutment distance was negatively correlated with the accuracy of photogrammetry and IOS. Differently from Carneiro Pereira, the distances were calculated between all pairs of abutments, making a direct comparison impossible.

### 3.3. Risk of Bias

**Figure 2:**
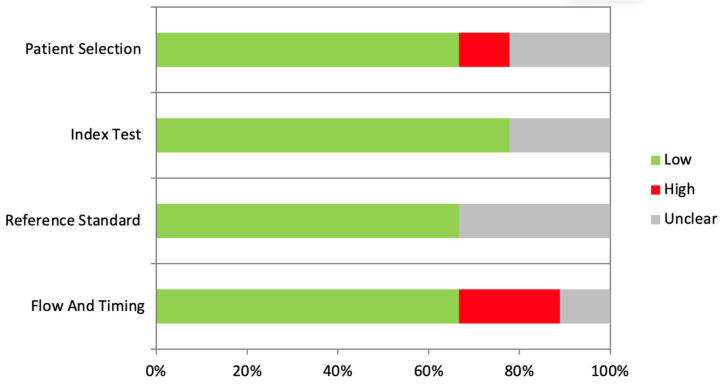
Graphical representation of the risk of bias.

The results of the risk of bias analysis are reported in Table 2 and Figure 2. The overall risk of bias was evaluated as low.

### 3.4. Quantitative Synthesis

Only three studies presented comparable data on the 3D accuracy of STL files and were therefore included in the meta-analysis [25,26].

Papaspyridakos et al. [26] compared the accuracy of full-arch impressions using conventional and digital techniques in 27 patients with 36 edentulous jaws (21 maxillary and 15 mandibular), all treated with one-piece, screw-retained implant-supported fixed complete dental prostheses. Both conventional impressions and intraoral digital scans were taken, with the resulting STL files analysed via reverse engineering software. They concluded that both impression techniques produced 3D deviations within clinically acceptable limits, with no significant accuracy difference between maxillary and mandibular jaws.

A similar analysis was conducted by Chochlidakis et al. [25]. Their study assessed the accuracy of full-arch digital impressions versus conventional impressions in 16 patients who received maxillary implant-supported fixed complete dentures. They identified a positive correlation that was not even statistically significant between the number of implants and 3D deviations. They concluded that the 3D accuracy of full-arch digital implant scans was within the clinically acceptable threshold.

Jasim et al. [23] compared the accuracy of conventional and digital implant-level impressions for atrophied maxillary ridges. Twelve participants with six implants each underwent two impression techniques: conventional (splinted open-tray) and digital. Accuracy was evaluated using two-dimensional and three-dimensional methods, as well as clinical assessments of framework passivity with the Sheffield test and radiographical assessment. Their findings were that the digital impressions had significantly greater deviations in both two-dimensional and three-dimensional accuracy compared to the conventional impressions and the digital impressions also showed a higher incidence of framework misfits.

**Figure 3:**
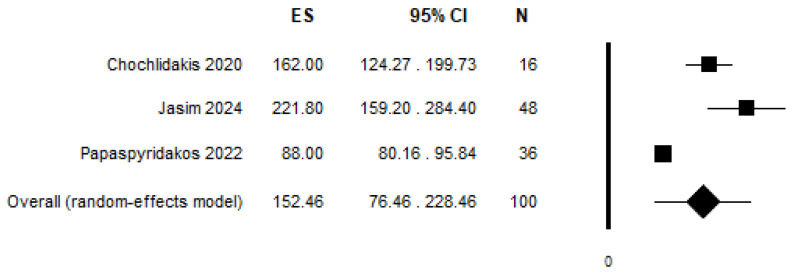
Results of random effects meta-analysis, pooling mean differences from the three included studies [23,25,26].

The random effects meta-analysis is reported in Figure 3 and resulted in a pooled mean difference of 152.46 (95% C.I. = 76.46–228.46, *p*-value < 0.001, I^2^ = 93.48%).

## 4. Discussion

The digital revolution has brought a new era in prosthodontics marked by the adoption of IOS [29], which might replace traditional analogic impressions [30]. These scanners have undergone significant advancements in recent decades, achieving remarkable precision in replicating the dental arch [9,31]. This systematic review seeks to investigate the accuracy of IOS in full-arch scanning of patients compared to analogic impression. One major challenge in conducting such studies lies in the absence of a standardised protocol for evaluating intraoral impression accuracy. While in vitro investigations can utilise instruments such as coordinate measuring machines or extraoral laboratory scanners to establish a reference model, replicating this approach in vivo is impossible.

Moreover, treating edentulous patients presents a particular challenge due to the absence of crucial anatomical landmarks essential for IOS referencing, especially in the mandible [32,33]. Conversely, in the maxilla, the presence of the palatal mucosa, along with the distinct palatine rugae, provides additional reference points for IOS scanning [34].

The results of the present review suggest an accuracy of intraoral scanning similar to the accuracy obtained with conventional impression. Nevertheless, it is important to highlight the significant heterogeneity observed among the studies included in this analysis (different IOS, scanning patterns, materials used for conventional impressions, etc.). Similar results were obtained by Ma et al. [34], who, in a systematic review, concluded that the accuracy of IOS impressions varies significantly based on the scanning approach, with trueness and precision in partial and full arches still uncertain. Follow-up clinical studies suggest IOS impressions are reliable in practice. However, these findings should be interpreted cautiously, as some data were derived from the same research group [34].

While five studies examined the fit of prostheses obtained through digital or conventional impressions, substantial variations in evaluation methodologies made a meta-analysis unfeasible. Analysing the radiographic gap between the frameworks and implant heads or multiunit abutments revealed that each author employed a different measurement approach. For instance, Roig et al. [4] categorised the gap into five classes, from 1 to 5, with 1 representing no gap and increasing at 0.15 mm increments until reaching 0.60 mm (score 5). De Angelis [21] measured the number of pixels and expressed the number in a linear function. Pera et al. [24] classified the passivity of the framework as excellent if the framework was found to be seated in place without any gap at the interface with the MUA/implant head or bad if the framework was not seated in place and presented gaps at the interface with the MUA/implant head. Gherlone et al. [22] just registered the presence or absence of a void at the implant/bar interface. It is the hope of the authors that, together with an evaluation of the accuracy of the STL, a clinical radiographic evaluation of the precision of the bar constructed based on conventional and digital impressions will also be made in future studies.

The other studies compared the STL files obtained both by digital impression and by digitalisation of the master cast created with conventional impression. Carneiro Pereira et al. [27] and Fu et al. [28] performed a linear assessment; however, the linear distance was measured in two different ways. The first one measured the linear displacements of each replica of the implants. The latter one measured the distances between all the pairs of abutment analogues based on the coordinates of the central point and the central axis.

To standardise the studies, the authors recommend measuring the linear differences between each abutment by comparing the STL file obtained from the digital impression with the one obtained with a conventional impression. This is a much more meaningful way than measuring the RMS deviation alone. The ideal would be to relate the error to the measured distance. For example, an error of 5 μm over a distance of 10 mm is more serious than an error of 5 μm over a 20 mm distance.

Cai et al. [35], in a systematic review, included clinical and in vitro studies reporting the accuracy of digital full-arch impressions. The primary outcome measured was the 3D deviations between the study reference models. They included 49 studies; 41 in vitro studies were meta-analysed. Eight clinical studies were discussed. Their results were that in studies using RMS, the results favoured IOS in the non-parallel situation with a mean difference of 99.29 μm (95% CI: [141.38, 57.19], I^2^ = 81%). Conversely, when implants were parallel, the results favoured conventional impressions with a mean difference of 13.62 μm (95% CI: [10.97, 16.28], I^2^ = 26%). For different brands of IOS, the accuracy ranged from 76.11 μm (95% CI: [42.36, 109.86]) to 158.63 μm (95% CI: [14.68, 331.93]).

It must be underlined that the study by De Angelis et al. [21] was the only one taking into consideration impressions and scans taken immediately after implant insertion and not in healed sites. While intraoral immediate scans might be more challenging (due to blood and unhealed flaps), in this study the accuracy outcomes were considered satisfactory.

### 4.1. Clinical Implications

The conclusions of the present systematic review present IOS as a clinically acceptable method to fabricate accurate fixed implant-supported prostheses in completely edentulous arches, particularly for non-parallel implants. However, a greater standardisation of the methods to measure accuracy is needed in clinical studies to improve knowledge on the topic and to make the results of different studies comparable. The precision of impressions, as an intermediate step in creating an optimal prosthesis, is a critical factor for the success of treatment. However, when comparing conventional and digital methods, this precision must ultimately align with clinical efficacy—namely, the clinical outcomes and the potential occurrence of biological and mechanical complications.

### 4.2. Limitations and Future Recommendations

The main limitation of the present research is the considerable heterogeneity of the included studies, which allowed only two studies to be included in the meta-analysis, reducing its significance. Additionally, the meta-analysis reports the mean differences between STL files obtained from digital impressions and those obtained by digitising casts produced through conventional impressions, increasing the risk of errors. Future research should focus on comparing linear deviations between conventional and digital impressions, not in absolute terms but by relating them to the measured distances. Additionally, information on mechanical problems must be registered.

## 5. Conclusions

In conclusion, the results of the present systematic review reveal contradictory findings regarding the accuracy of digital impressions. However, most studies analysing the clinical performance of prostheses obtained through digital impressions suggest that their accuracy falls within clinically acceptable thresholds. It is important to emphasise the significant heterogeneity among the included studies.

## Figures and Tables

**Table 1:** Main data for the included articles.

Study (Author and Year)	Methodology	Number of Patients	Number of Implants	Max/Mand	IOS	Conventional Impression	Parameters Evaluated
Roig et al., 2022 [4]	Prosthesis evaluation	12	5–7 For a total of 78	Maxillary	TRIOS 3	Polyether: Impregum, 3M ESPE	Radiographic fit The Sheffield test
Gherlone et al., 2016 [22]	Prosthesis evaluation	25	4 For a total of 120	17 Maxillary 13 Mandibular	TRIOS 3	Polyether: Permadyne, ESPE	Radiographic fit The Sheffield test
De Angelis et al., 2023 [21]	Prosthesis evaluation	150			TRIOS 3	PVS	Radiographic fit The Sheffield test
Pera et al., 2023 [24]	Prosthesis evaluation and STL data comparison	9	4–6 implants For a total of 51	6 Maxillary 1 Mandubular	MACH2	White Plaster, Ker	Radiographic fit The Sheffield test The standard deviation of discrepancies among the STL files
Chochlidakis et al., 2020 [25]	STL data comparison	16	4–6 implants		True Definition	PVS	3D implant deviations
Papaspyridakos et al., 2023 [26]	STL data comparison	27	4–6 implants For a total of 207	21 Maxillary 15 Mandibular	TRIOS 3	Polyether	3D implant deviations and the root mean square
Carneiro Pereira et al., 2022 [27]	STL data comparison	10	4 implants For a total of 40	Mandibular	TRIOS 3	Splinted impression copings with acrylic resin	The distance between the implants, implant linear displacements, total 3D displacements and angle projections
Fu et al., 2023 [28]	STL data comparison	15	115 implants	9 Maxillary 13 Mandibular	TRIOS 3	PVS	Distance and angle between the abutment analogues and the root mean square (RMS)
Jasim et al., 2024 [23]	Prosthesis evaluation and STL data comparison	12 participants	6 implants in each maxilla	12 Maxillary	Medit I-500	PVS	Linear displacements, total 3D displacements. Radiographic fit The Sheffield test

**Table 2:** Risk of bias.

Study	Patient Selection	Index Test	Reference Standard	Flow and Timing
Roig et al., 2022 [4]	-	-	-	-
Gherlone et al., 2016 [22]	?	-	?	-
De Angelis et al., 2023 [21]	-	-	-	-
Pera et al., 2023 [24]	-	-	-	-
Chochlidakis et al., 2020 [25]	-	-	-	?
Papaspyridakos et al., 2023 [26]	+	?	?	+
Carneiro Pereira et al., 2022 [27]	?	?	?	+
Fu et al., 2023 [28]	-	-	-	-
Jasim et al., 2024 [23]	-	-	-	-
